# An Evidence-Based Review: The Effects of Malaysian Traditional Herbs on Osteoporotic Rat Models

**DOI:** 10.21315/mjms2018.25.4.2

**Published:** 2018-08-30

**Authors:** Nur Adlina Mohammad, Norfarah Izzaty Razaly, Mohd Dzulkhairi Mohd Rani, Muhammad Shamsir Mohd Aris, Nadia Mohd Effendy

**Affiliations:** Basic Medical Sciences, Faculty of Medicine and Health Sciences, Universiti Sains Islam Malaysia, Menara B, Persiaran MPAJ, Jalan Pandan Utama, Pandan Indah, 55100 Kuala Lumpur, Malaysia

**Keywords:** phytochemicals, computed tomography, bone density, osteoporosis, bone fractures, anti-oxidant

## Abstract

Osteoporosis is considered a silent disease, the early symptoms of which often go unrecognised. Osteoporosis causes bone loss, reduces mineralised density, and inevitably leads to bone fracture. Hormonal deficiencies due to aging or drug induction are also frequently attributed to osteoporosis. Nevertheless, the phytochemical content of natural plants has been proven to significantly reduce osteoporotic conditions. A systematic review was conducted by this study to identify research specifically on the effects of Malaysian herbs such as *Piper sarmentosum, Eurycoma longifolia* and *Labisia pumila* on osteoporotic bone changes. This review consisted of a comprehensive search of five databases for the effects of specific herbs on osteoporotic bone change. These databases were Web of Science (WOS), Medline, Scopus, ScienceDirect and PubMed. The articles were selected throughout the years, were limited to the English language and fully documented. Duplication, irrelevant titles, different herbs and in vitro studies were excluded, including those that are not original research papers. A total of 399 potential studies were identified, but only 21 samples were accepted based on the inclusion and exclusion criteria. Six of the twenty one studies were on *Piper sarmentosum,* six on *Eurycoma longifolia,* and the remaining nine studies were on *Labisia pumila*. Overall, in three of the studies a glucocorticoid-induced model was used, while in 12 of the studies an ovariectomised model was used, and for the other six studies an orchidectomised model was used as the osteoporotic model. All of the studies reported varied results based on the type of herbs used, but in comparison to *Eurycoma longifolia, Piper sarmentosum* and *Labisia pumila* recorded better anti-osteoporotic effects, while the majority of studies on *Eurycoma longifolia* were unable to preserve bone strength.

## Introduction

Osteoporosis is a systemic skeletal disease. It compromises bone strength and microarchitecture, which becomes clinically evident when fractures occur. The World Health Organization (WHO) defined osteoporosis as low bone mineral density that lies 2.5 standard deviations or more below the average bone mineral density of a young adult female (1). About 1.3 million to 1.7 million worldwide cases of hip fracture were estimated in 1990 and this is estimated to rise to 3 million in 2025 (2). Osteoporosis is three times more common in women than men, but the mortality risks after fracture are higher for men than for women (3). This increment makes osteoporosis an economic burden in terms of medical provision, as well as limiting the individual’s work capabilities.

Osteoporosis has been categorised into primary and secondary osteoporosis. Primary osteoporosis is associated with hormone deficiency and secondary osteoporosis is a consequence of secondary effects from other diseases or drugs usage (4). Primary osteoporosis is more prevalence in both male and female populations than secondary osteoporosis, which is recorded above 50% out of 369 patients recruited (5). Estrogen and androgen play a crucial role in skeletal development during growth state and are involved in skeletal homeostasis throughout adulthood (6). Reduction in estrogen levels causes an increase in bone remodeling activities, which consequently leads to bone loss in both women and men. Estrogen deficiency causes depletion in the number of osteoblasts in the bones by inhibiting maturation at a cellular level while enhancing the synthesis of cytokines which play roles in bone resorption (7). Depletion of estrogen increases the bone resorption activity of osteoclasts higher than bone formation of osteoblast, inevitably leading to bone deterioration. Even though estrogen is common among women, it is also an important consideration for ageing men, as androgen gradually fails to be converted to estrogen which is derived from testosterone aromatisation (3, 6). Long-term glucocorticoid therapy is a common contributor to secondary osteoporosis. Glucocorticoid induces osteoporosis by interfering Wnt signaling pathway in regulating osteoblast function and stimulates osteoclast development (8). In vivo osteoporosis studies are primarily assessed using an ovariectomised model for estrogen-deficiency factor, an orchidectomised model for androgen-deficiency factor, and by glucocorticoid-induction for drug-related osteoporosis.

Hormone replacement therapy (HRT), either using estrogen or testosterone, is the main treatment for treating or reducing occasions of osteoporosis which are due to hormone deficiency. Sexual hormones are believed to protect bone remodeling from oxidative stress, where hormone withdrawal alters the generation of reactive oxygen species (ROS) (9). ROS stimulates the bone resorption process by activating osteoclastogenic Nuclear Factor-kappa *β* (NF-k *β*) ligand (RANKL), and macrophage colony-stimulating factor (M-CSF) which initiates osteoclast differentiation and activation (9, 10). Oxidative stress may cause melondialdehyde (MDA) levels to increase and may inhibit anti-oxidative enzymes such as superoxide dismutase (SOD) and glutathione peroxidase (GPx) levels (10). Although effective, HRT has been proven to cause significant adverse effects such as muscle spasms, nausea, heart attacks, breast cancer, and strokes (11). Alternatives such as *Piper sarmentosum* (*Kaduk*), *Eurycoma longifolia* (*Tongkat Ali*) and *Labisia pumila* (*Kacip Fatimah*), which are widely known herbs in Southeast Asia, especially in Malaysia, have therefore been studied by researchers for their therapeutic effects against osteoporosis.

*Labisia pumila* is a herbaceous plant with creeping stem which belongs to *Myrsinaceae* family (12) is known by the locals as *Kacip Fatimah* and *Selusuh Fatimah*. In Malaysia, there are three varieties: *L. pumila* var*. pumila, L. pumila* var*. alata,* and *L. pumila* var*. lanceolata* (13). *L. pumila* var. *alata* has a winged petiole and red veins, while var. *pumila* has a marginate petiole and an ovate leaf blade shape, and var. *lanceolata* has a long and non-winged petiole (13). *L. pumila* has been utilised by Malay women for many decades in their daily routine for improving their general well-being and vitality, maintaining their figure and appearance, assisting with the reduction of hormonal imbalance symptoms, as well as for generally increasing their libido (14). The total flavonoid and phenolic content in *Labisia pumila* extract contributes to its therapeutic properties in anti-inflammatory, anti-microbial, anti-oxidant, and anti-cancer treatments; all indicating the possibility that this species could also help in the treatment and prevention of osteoporosis (15). *L. pumila* also possesses phytoestrogenic properties, being able to mimic estrogen and bind to estrogen receptors, therefore exerting a weaker estrogenic effect compared to endogenous estrogen. Moreover, it is able to manipulate steroid-hormone stimulation through the sex-hormone binding globulin (SHBG) and by displacing estradiol and testosterone (16).

*Eurycoma longifolia* or *Tongkat Ali* is a tall, slender, shrubby tree which grows in sandy soil and the name represents its long twisted root (17). *Eurycoma longifolia* is widely known among Malaysians, being especially used by men as herbal supplement, either in capsulated or powder form. It is reported to help with enhancing health and libido in men (18). A variety of chemical compounds have been isolated from *E. longifolia* roots, including eurycomaside, tannins, high molecular weight polysaccharides, glycoproteins, mucopolysaccharides and alkaloids (19). *E. longifolia* also contains the bioactive compound eurycomanone, which may indeed influence testosterone production via the hypothalamic-pituitary-gonadal axis, where it induces the release of FSH and LH into the circulatory system and promotes the Leydig cell in the testes to produce testosterone (18).

*Piper sarmentosum* is a herbaceous plant from the family *Piperaceae* which is widely distributed in tropical and sub-tropical regions such as Thailand, Malaysia, the Philippines, China, and Vietnam. *P. sarmentosum* is a slender, aromatic, perennial climber, with woody roots and numerous wide ovate, cordate leaves (20). It is known by the locals as *Kaduk*, *Chau Pulu* or *Jia Jau* (21). It has been used for a long time as natural remedy for a series of diseases like malaria, toothache, dysentery, cough, flu, pleurisy and backache (22). The bioactive compound found in *P. sarmentosum* includes alkaloids, amides, pyrones, dihydrochalcones, flavonoids, phenylpropanoids, lignans and neolignans (23). Previous investigations reported that naringenin is one of the flavonoid groups that possesses high free-radical scavenging activity which contributes to its capability in improving bone strength and preventing or reducing the bone loss of ovariectomised rat models (24–26).

Nowadays, traditional herbs are still used extensively, even in a rapidly developing country such as Malaysia. Herbal medicines are widely preferred due to their known effectiveness, fewer side effects, and relatively low cost compared with the synthetic drugs which are associated with many risky effects such as stroke, heart attack, and cancer. According to Abd Jalil et al. (3), many scientists and doctors have become interested in traditional medicines because of their medicinal potential in treating some serious disease such as osteoporosis, as well as to avoid adverse effects of synthetic drugs. It is also the conviction of this study that research on natural plants or herbs as alternative medicine should be explored comprehensively. The aim of this systematic review is to assess the original research articles to determine the efficiency of the selected herbs in preventing bone loss of osteoporotic rat models.

## Search Results

The primary objective of this review was to assess the efficiency of selected traditional medicinal herbs as alternatives to hormone replacement therapy against osteoporosis. This review specifically targeted research results conducted in vivo, while also highlighting the use of some experimental tests. The databases used in this study include Medline, Scopus, Pubmed, ScienceDirect and Web of Science (WOS). The search strategy was to track the keywords as follows:

1. (*Labisa pumila* or *Kacip Fatimah*) and (osteoporo* or ovariecto* or bone loss)
2. (*Eurycoma longifolia* or *Tongkat Ali*) and (osteoporo* or orchidecto* or bone loss)
3. (*Piper sarmentosum* or *Kaduk*) and (osteoporo* or ovariecto* or orchidecto* or bone loss)

### Selection of Research Articles

The results were limited to fully documented articles which were published in the English language with no limitation on the date of publication. Studies which fell within the inclusion criteria were as follows: i) animal studies which relate to osteoporosis, ii) where the intervention subject is among the medicinal herbs stated in the keywords. Studies may include papers in proceedings. The studies were excluded if they were: i) duplicated studies; ii) reviews, news, or case studies; iii) not related to bone metabolism analysis; and iv) irrelevant titles.

### Identification of Relevant Studies and Data Extraction

Manuscripts in this review were selected through the inclusion criteria based on their titles. Next, the abstract of the remaining papers were screened and any papers that did not fit the inclusion criteria were excluded. Lastly, the full text of the papers was screened and those which did not fulfill the inclusion criteria were removed. The final data were again screened by two reviewers for the data extraction phase. We summarised and recorded the data as shown in Table 1, Table 2 and Table 3, according to the type of herb. Categorisation was based on i) type of extraction; ii) type of analysis/tests used; iii) a brief description of samples; iv) a brief description of methodology; v) a brief explanation of the results and vi) outcomes and comments on the study.

### Search results

**Figure 1:**
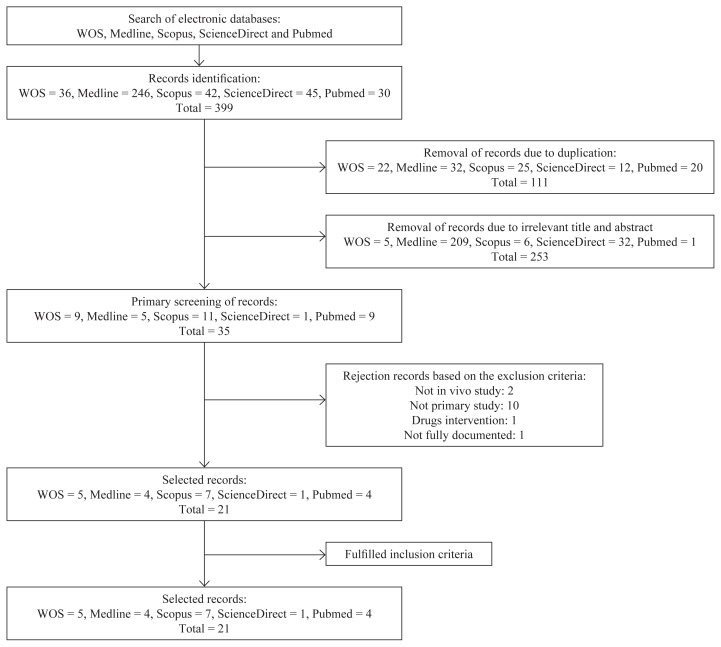
Flow chart of the selection process of the articles used in this review

The literature searches identified 399 potentially relevant articles. A total of 288 articles were then selected, after removing duplicate papers. After further scrutiny of the relevancy of the titles and the abstracts, 35 articles were accepted. Finally, 21 studies which fulfilled all of the inclusion and exclusion criteria were used in this review. Figure 1 illustrates the article selection based on the inclusion and exclusion criteria.

### Study characteristics

All of the studies were in fact conducted on animals and appeared in the literature between 2009 and 2017. Rat models were used exclusively; specifically 15 studies used Sprague-Dawley and the remaining used Wistar rats. Most of the female rats were above three months and weighed between 200 g and 250 g and the male rats were mostly over 10 months old and weighed between 300 g and 500 g. Three types of osteoporosis model were the focus of this review: glucocorticoid-induced osteoporosis, androgen-deficiency osteoporosis and estrogen-deficiency osteoporosis. Three studies on glucocorticoid-induced osteoporosis were conducted on adrenalectomised rats, six studies used orchidectomised models for investigation of androgen-deficiency osteoporosis and 12 studies used ovariectomised models for investigation of estrogen-deficiency osteoporosis. Treatments were primarily administered for six weeks to nine weeks, excluding incubation and acclimatisation periods. The minimum sample size used was 24 (*n* = 24) and the maximum was 96 (*n* = 96).

This paper reviewed the anti-osteoporotic effects of herbs. There were six studies on *Piper sarmentosum,* six studies on *Eurycoma longifolia,* and nine studies on *Labisia pumila.* The therapeutic effects of these herbs were tested in terms of bone biomechanical strength, bone biochemical markers, bone microarchitecture, bone histomorphometry, bone-related gene expressions and bone callous radiograph stages. Overall, seven studies focused on bone biochemical, six on bone biomechanical tests, five on bone microarchitecture, three on histology, two on gene expression, and one study was on bone callous stages. Bone biomechanical analysis were undertaken using the Instron Microtester 5848 Model, bone microarchitecture via micro-CT (*μ*CT 80 scanner, Scanco Medical), and bone callous stage via CT scanning and X-rays. Whereas bone biochemical analysis was performed mostly using an enzyme-linked immunosorbent assay (ELISA) test which included the bone markers, bone oxidative status, calcium content, serum cortisol level, testosterone level, corticosterone level and 11*β*-HSD type 1 expression. Histological analysis was performed using hematoxylin and eosin (H&E) staining and the Von Kossa staining methods.

## Assessments of Bone Metabolism of Osteoporotic Rats Model

Bone biochemical analysis is the primary analysis used in this review. A total of 4 out of 21 studies examined bone resorption markers of osteocalcin and collagen type 1 cross-linked C-telopeptide (CTX-1) using the Rat Osteocalcin ELISA and the RatlapsTM ELISA CTX-1 kit. Calcium content analysis was carried out in two studies using an atomic absorption spectrophotometer. Furthermore, two studies were conducted on 11β-hydroxysteroid dehydrogenase (11*β*-HSD) type 1 expression and were tested via the *Rattus norvegicus* ELISA Kit and dehydrogenase activity assay, along with photomicrograph analysis. Only one study out of twenty one identified bone oxidative status based on the superoxide dismutase enzyme (SOD) via the Superoxide Dismutase Assay Kit, gluthathione peroxide (GPx) via the Gluthathione Peroxidase Assay Kit, and malondialdehyde (MDA) via the thiobarbituric acid reactive substances (TBARS) Assay Kit. Only one study identified the serum testosterone level of the androgen-deficient rats.

Bone strength is key to understanding the fracture risk associated with low bone mineral density. A fracture is a structural failure of the bone where the forces applied to the bone exceed its load-bearing capacity (27). Six of the studies measured bone mechanical strength using the Instron Microtester 5848 Model to identify bone strength physically on the following structural parameters: maximum load, stress, strain, and Young’s modulus, which are called the three point bending test. There are two categories of bone mechanical strength; extrinsic and intrinsic. Extrinsic parameters reflect the properties of the whole bone which are affected by various external factors which include load, displacement, and stiffness. Intrinsic parameters, in contrast, refers to the inner material of the bone such as their geometric distribution and cellular metabolic activity affecting the bone to bear loads (16). Intrinsic parameters include stress, strain, and Young’s modulus or elasticity. Maximum load is defined as the amount of load that the bone can sustain before it suffers permanent damage, which indicates the point where the femur starts to change from elastic to plastic (16, 18). Increment of load indicates that the bone is stronger. Displacement is defined as the length of deformation that the bone can sustain before failing. It is inversely related to the brittleness, used to measure bone ductility. Stress is the point at which plastic deformation begins if the stress is beyond the yield point and bone deformation becomes plastic (28), while strain refers to the relative deformation of the bone before it re-fractures (29). Young’s modulus, or elasticity, is the slope of stress-strain curve that represent the stiffness of the material; the higher the elasticity, the stiffer the bone (30).

Bone histomorphometry is a method used to quantify any bone mineralisation defects, otherwise known as the conventional method for measuring the bone’s structural parameters in two-dimensional information. Three histomorphometry studies were carried out by Estai et al. (31), Ariff et al. (32) and Fathilah et al. (33). A histological test was administered by Estai et al. (31) using H&E staining to observe the fracture healing score measured by Allen’s grading system, whereas Ariff et al. (32) and Fathilah et al. (33) used the Von Kassa staining method in their study. The study examined the bone characteristics of structural parameters, which are: trabecular volume, thickness, number and separation; static parameters of osteoblast surfaces, osteoclast surface, eroded surface, osteoid volume and osteoid surface; and dynamic parameters of single-labeled surface, double-labeled surface, mineralising surface, mineral apposition rate and bone formation rate. Basically, the procedure of histomorphometry involves three parts for each parameter. The femora were dissected into two divisions. One half was embedded in methyl methacrylate, sectioned, and then stained using the Von Kassa method, before being analysed under a microscope. The stained parts represent structural parameters, while the unstained parts indicate dynamic parameters. The other halves of the femora were decalcified with ethylene diamine tetra acetic acid (EDTA), embedded in histological wax, prepared as histological slides and then analysed under a microscope.

Micro-CT is a currently used methodology which overcomes the limitations of conventional 2D histomorphometry. The imaging of specimens, bone biopsies, and small animal investigations are mostly done by micro-CT scanners. Micro-CT analysis provides 3D images which help to identify any topographic changes which have occurred within the healing bone, such as the compact of callous and the trabecular network (34). This analysis is useful in assessing the effects of pharmacological compound on the bone. This review records five studies which used a *μ*CT80 scanner (Scanco Medical, USA) to evaluate the bone microarchitecture, which consists of metric parameters of bone volume, trabecular thickness, trabecular number and trabecular separation. The non-metric parameters include connectivity density, structural model index and degree anisotropy. The *μ*CT80 scanner used in the studies was set at an energy source of 70KVp and 11A*μ*A, with high 0.5 mm filter and high resolution (35–39). Also, a study conducted by Estai et al. (25) investigated the bone callous stage in the area of fracture healing cases by grading the stages using a 5-point radiograph-based scoring system. A CT scanner and an X-ray machine were used.

## Therapeutic Effects of *Piper Sarmentosum* against Osteoporosis

As stated previously, six studies used *Piper sarmentosum* (PS) extract as a therapeutic supplement against osteoporosis. Of these, three studies were assessed through bone biochemical analysis (Asri et al. (26); Ima-Nirwana et al. (40); Ramli et al. (41)), one study through bone biomechanical analysis (Estai et al. (24)), one analysed bone callous stages (Estai et al. (25)) and one was a histological study (Estai et al. (31)).

The effects of PS on bone callous strength were initially evaluated by Estai et al. (25), who used an ovariectomised model. The axial callous volumes of the fractured bones were measured using a CT scan. A higher callous volume would indicate a delay in fracture healing. Estai et al. (25) reported that the results of the bone callous stage analysis via a radiological study of the PS supplemented rats was comparable to standard estrogen replacement: both were able to retain a score as good as the Sham group level. In the same year, these researchers supported their finding by a histological analysis which reported that the median fractured callous healing of the group treated with PS was higher than the negative control group and identical to standard estrogen replacement treatment. The stained callous structure of rats treated with PS was similar to that of the Sham group. Their latest study in 2012 was also able to prove that the physical strength of healed fractured bones which had been treated with PS had a higher maximum load, stress, and Young’s modulus than the negative control group and at par with the Sham and the standard estrogen replacement therapy groups. This review supports the conclusion that the therapeutic effects of PS are more promising against estrogen-deficiency osteoporosis than against glucocorticoid-induced osteoporosis. This may be due to its phytoestrogenic and anti-oxidative properties.

Systemic, or long term use of glucocorticoid is proven to cause a reduction of bone mineral density due to the effects of insufficient matrix production by osteoblasts, impaired matrix mineralisation, increased osteoblasts and osteocyte apoptosis, and the prolonged life-span of osteoclasts (42). Weinstein (43) mentioned that glucocorticoid administration is the most common known factor of secondary osteoporosis. A study reported that ageing mice were positively correlated with the adrenal production of glucocorticoids and 11β-hydroxysteroid dehydrogenase (11*β*-HSD) type 1 induction (44). 11*β*-HSD type 1 is an enzyme found in bone which activates glucocorticoid activity. The inhibition of 11*β*-HSD type 1 may possibly treat the metabolic disorder. Findings by Ramli et al. (41) found that the supplementation of PS to adrenalectomised rats significantly reduced the 11*β*-HSD type 1 expression compared to the negative control group. Another study found that, even though the 11*β*-HSD type 1 expression was reduced, rats supplemented with PS did not show any significant differences when compared to the negative control rats (26). He added that there were no significant differences in both the serum and the femur corticosterone levels between all groups. Ima-Nirwana et al. (40), nevertheless, concluded that PS could be a potential anti-osteoporotic agent, as it showed significantly equal results to standard glycyrrhetinic acid (GCA) treatment. In fact, after eight weeks of treatment, the serum cortisol of PS was maintained at Sham level, and the bone resorption marker level was significantly decreased to below the adrenalectomised control group.

## Therapeutic Effects of *Eurycoma Longifolia* against Osteoporosis

Six studies evaluated the effects of *Eurycoma longifolia* (EL) on the androgen-deficiency osteoporosis of orchidectomised rat models. Two out of the six studies exclusively investigated bone microarchitecture (Azri et al. (35), Ramli et al. (36)); one was a biomechanical strength study (Azri et al. (18)). The remaining were a combination of tests on bone microarchitecture, biomechanical strength biochemical markers, histomorphometry, and gene expression (Shuid et al. (37); Ariff et al. (39); Saadiah et al. (45)).

Osteoprotegerin (OPG), RANKL, and M-CSF expression are other factors which contribute to the activation of bone resorption. OPG is known as an osteoclastogenesis inhibitory factor that is produced by osteoblasts working to prevent the activation of RANK and to inhibit any osteoclast formation (46). The inhibition of RANKL suppresses bone resorption and increases cortical and cancellous bone volume, density, and strength (47). At the same time, the M-CSF released by osteoblasts will bind to receptors on the osteoclast and initiate osteoclast differentiation, activation, and survival (48). A study conducted by Shuid et al. (37) reported that EL was able to increase the OPG expressions of osteoporotic bones similar to the Sham level, while decreasing the M-CSF expressions. This was a significantly similar effect to standard testosterone replacement, while the RANKL expression was also significantly consistent between all the groups. The EL extract was, therefore, only found to affect OPG expression, which may be an additional mechanism of EL in protecting against bone resorption induced by androgen-deficiency.

A 3D image of bone microarchitecture produced by a CT scan is widely used in evaluating the level of bone porosity and the stage of bone healing. A bone microarchitectural analysis which was carried out by Ramli et al. (36) found that the 3D images of the trabecular microstructure of EL treated groups seemed to be more porous than those of an orchidectomised-control group. In addition, the loss of trabecular bone connectivity for those groups was more apparent compared to the Sham and testosterone treatment groups. Even though EL treated groups recorded a significantly higher trabecular separation and a significantly lower trabecular number compared to the Sham and testosterone treatment groups, the group which supplemented with 90 mg/kg of EL extracts showed a significantly more positive result in preserving bone volume. Recent studies by Azri et al. (35) reported that the 3D images of refractured healed bones of an EL group showed better bridging cortical connections compared to the bones of orchidectomised rats, but more inferior to the Sham and standard testosterone replacement treatment groups. In addition, the fraction of mineralised callouses was increased but not significantly different compared to that of the negative control group. The measures of bone mineral density and soft callous volume between all the groups were not significantly different. Histomorphometry studies by Ariff et al. (39) also corroborated the bone microarchitecture analysis, as both of the results did not show any significant difference in trabecular thickness and separation between the EL administrated group and the negative control group. It can be concluded that EL may improve fractured bones only by increasing bone volume but that it has no effect on other bone trabecular parameters.

The three point bending test evaluates bone strength in term of bending breaking force (breaking or fracture load), stiffness, ultimate stress (breaking strength), and elastic modulus; all of which are good indicators of the mechanical strength of cortical bone (49). The test demonstrated that the bone strength of the EL treated group was not significantly improved in comparison with the orchidectomised group. The EL treatment did not show any significant difference in the testosterone replacement group, except for the maximum stress parameter (18). Another study by Saadiah et al. (45) also reported that EL supplementation did not improve bone strength statistically, except when the supplementation of EL was mixed with testosterone.

Bone marker analysis is one of the most important assessments of bone metabolism such as osteocalcin and CTX-1. Osteocalcin is a bone marker synthesised in the skeleton by osteoblasts; its elevation indicating the increasing osteoblast activity of bone formation (50). Beta-C-terminal telopeptide (CTx) is a peptide fragment that is released by osteoclasts and is found to be increased in hormone deficient rat models, indicating high bone resorption activity (37, 51). The serum osteocalcin level of rats treated with EL was not significantly different from the other groups but had potential to lower the CTx level better than in the Sham and orchidectomised groups, albeit it not to a significant level (Shuid et al. (37)). Saadiah et al. (45) also reported similar findings but they added that only a mixture of EL with testosterone supplementation was able to significantly increase the osteocalcin level compared to the Sham group. Furthermore, the serum testosterone level and bone calcium content of the EL treated group showed no significant difference compared to the orchidectomised control group, although the levels were shown to be increasing (37, 32). It can therefore be generally concluded that EL is not an effective agent in preserving androgen-deficiency osteoporotic bones. This limitation is probably due to the total removal of the testes, which are, of course, the main factor in the production of testosterone. Certain studies recommend the use of orchiectomised models instead of orchidectomised or drug-induced osteoporosis such as glucocorticoid, thyroxine, aromatase inhibitor and thiazolidinediones (52).

## Therapeutic Effects of *Labisia pumila* against Osteoporosis

There were a total of nine studies on the effects of *Labisia pumila* (LP) on bone metabolism. A total of three studies were on bone microarchitecture by Nadia et al. (38, 39, 53), two studies were on bone mechanical strength (Fathilah et al. (54); Nadia et al. (16)), while the remaining three were on bone biochemical makers (51), bone oxidative status (55), gene expression (56), and histomorphometric analysis (33).

The first study on the effects of LP on bone microarchitecture was conducted by Nadia et al. (39) using a *μ*CT80 scanner (Scanco Medical, USA). They documented that rats fed with 100 mg/kg and 20 mg/kg of LP extracts showed a denser trabecular network than Sham and baseline groups. In fact, they found that nine weeks of 100 mg/kg of LP supplementation was more effective than estrogen replacement treatment. LP was able to significantly increase the bone volume and connectivity density, and significantly decrease the trabecular separation to a level lower than the ovariectomised control group after six and nine weeks of LP supplementation. Therefore, they concluded that *Labisia pumila* has great potential as an effective treatment in reversing ovariectomy-induced bone changes. Later in 2017, Nadia and her co-workers (38) went on to investigate which type of extractions (aqueous, methanol or ethanol) of LP was the best. They discovered that extracts of LP in an aqueous form were the most effective in preserving bone microarchitecture. Rats supplemented with an aqueous extract of LP had the densest microarchitecture, a better result than with the Sham group. Even though there was no significant difference in trabecular thickness, aqueous extracts of LP recorded significant increases in bone volume and connectivity as well as significantly lower trabecular separation compared to the ovariectomised-control group. This group also recorded better results within those parameters compared with other types of extraction. Therefore, it can be claimed with confidence that the aqueous extract of LP was found to be the best regiment in improving bone trabecular network in estrogen-deficiency osteoporosis.

The effects of LP supplementation on bone strength was assessed through the three point bending test using an Instron Microtester 5848 Model. Nine weeks of supplementation with aqueous extract of LP proved to be effective in improving the maximum load, displacement, stiffness, and Young’s modulus, especially for those treated with a 100 mg/kg concentration (39). Previously, Fathilah et al. (54) had found that LP was more effective than estrogen replacement therapy in preventing estrogen-deficiency osteoporosis. They reported that load, stress, strain, and Young’s modulus of LP treated rats was significantly similar to rats treated with estrogen and were significantly higher than the Sham and positive control groups. Fathilah et al. (33) further investigated the effects of LP on the trabecular bone network. They reported that LP extract was statistically identical to standard estrogen replacement in all structural (trabecular volume, thickness, number and separation), static (osteoblast surface, osteoclast surface, eroded surface, osteoid volume and osteoid surface) and dynamic (single-labeled surface, double-labeled surface, mineralising surface, mineral apposition rate and bone formation rate) parameters. It can therefore be concluded that LP supplementation was effective in improving bone strength due to the increase of the trabecular network.

Bone formation and resorption makers are key to determining the effectiveness of particular supplementations for bone regulation. The two main bone markers involved are osteocalcin and CTX-1. Osteocalcin levels reflect bone formation, whereas CTX-1 reflects bone resorption. The osteocalcin and CTX-1 levels of rats that were treated with LP aqueous extract were found to be significantly higher compared to same levels in the ovariectomised control group, and to a similar level in the estrogen replacement group (51). Another study conducted by Nadia and Shuid (55) reported that LP was capable of increasing anti-oxidative enzymes (SOD, MDA and GPx levels) in the bones of estrogen-deficiency postmenopausal rats. Both concentrations of 20 mg/kg and 100 mg/kg of LP extracts significantly increased the SOD level to a higher level than with the standard treatment group. The GPx level was found to be significantly increased by LP 100 mg/kg concentration higher than in the positive and baseline groups, and to a significantly similar level in the standard treatment group. The MDA of the treatment groups was significantly lower than the positive control groups, which means that LP was responsible for reducing lipid peroxidation activity in the bones of ovariectomised rats. LP supplementation exerts promising therapeutic effects on bones due to the anti-oxidative properties of its bioactive compounds that are capable of maintaining a balanced bone turnover activity.

Gene expression studies have reported that the RANKL expression of ovariectomised groups was increased, while the OPG and bone morphogenetic protein 2 (BMP-2) expressions were decreased. BMP-2 was used in reference to bone healing, as it acts to induce immature cells to differentiate into osteoblasts (57). Rats treated with LP extracts showed similar effects to those which had undergone estrogen replacement treatment, while both were able to restore OPG and BMP-2 levels, and to reduce the RANKL expression to the same level as the Sham group (56). The effects of LP on bone microarchitecture, biomechanical strength, bone markers and molecular expression indicate that *L. pumila* is as effective as conventional estrogen replacement therapy in improving the bone metabolism. It can, therefore, be confidently recommended as an alternative anti-osteoporotic agent to prevent and treat osteoporosis. It is also clear that this anti-osteoporotic effect of LP is due its bioactive compounds such as phenolics, flavonoids, and saponins, which contribute to its phytoestrogenic and anti-oxidative properties.

## Strengths and Limitations

Osteoporosis is a silent disease which only receives attention when fracture occurs. Although there are many conventional drugs to treat osteoporosis, they are known to cause adverse effects such as breast cancer, stroke and heart attack, so seeking for natural based medicines with equal effectiveness and fewer adverse effects is imperative. *Piper sarmentosum, Eurycoma longifolia* and *Labisia pumila* are all well-known Malaysian herbs which have, for many years been broadly recognised as herbs which have anti-malarial, anti-oxidant and anti-inflammatory properties but there is a notable lack of studies of their efficacy for osteoporotic bone improvement. A critical review is therefore relevant to identify the related published research papers and to analyse their potential as anti-osteoporotic agents. Following a thorough reading of the literature, it is clear that at the time of writing this is the first systematic review which focuses on the effectiveness of famous Malaysian herbs on osteoporotic healing.

There were some limitations for this study. Firstly, some studies did not mention what type of extractions were used. Different types of extraction may have influenced the outcome of bone metabolism results. In addition, the age of the rats used in some studies was not mentioned specifically. It is important to state the age, since the majority of studies involving hormonal-deficiency osteoporosis mostly occur due to the aging process. The osteoporotic area is also not uniform; some studies were on hormonal effects and others were on drug induced effects. In addition, the type of osteoporotic bone also varies, where some studies examined the healing process of fractured bones, while others focused on naturally occurring bone deterioration due to aging or hormonal deficiency.

## Recommendations

Based on this review, it is suggested to further investigate the effectiveness of the herbs in this study, which may have real consequences for health studies in this important and often overlooked area. Wider exploration of the potential of other natural plants or herbs for preventing osteoporosis are strongly recommended. Lastly, it has become clear from this study that it is very important for researchers to elaborate in detail on the type of extraction used in order to see what kind of extraction has better results, in order to ensure the validity of repeated experiments.

## Conclusion

This review concludes that the Malaysian herbs, *Piper sarmentosum, Eurycoma longifolia* and *Labisia pumila* shows very promising anti-osteoporotic potential. Even though the findings of the studies on *Eurycoma longifolia* are quite compromising compared to the others, it still recorded a small potential for preventing bone loss. A suitable model should also be selected, such as an orchiectomised or drug induced model, instead of an orchidectomised model. It can be conclude that naturally based medicines do have promising health effects, fairly like conventional drugs but with the benefits of less adverse effects. Further studies on the therapeutic effects of other natural plants against osteoporosis are warranted in order to provide a mechanistic overview of the anti-osteoporotic properties of these alternative agents.

## Figures and Tables

**Table 1:** Review of the effects *Piper sarmentosum* (PS) on the bone study

	Type of extraction	Type of study	Samples/populations	Methodology	Results	Comment/outcomes
**Study 1** **Asri et al. 2016** (26)	Aqueous extract	Bone biochemical analysis	250 g–300 g Sprague-Dawley (*n* = 24)	Rats were randomly grouped into: Sham with olive oil and normal saline (*n* = 8) Adrx with dexamethasone, 120 μg/kg and normal saline (*n* = 8) Adrx+PS with dexamethasone, 120 μg/kg and PS extracts 125 mg/kg (*n* = 8) The treatments were given once a day, six days a week for eight weeks. The serums were collected the day before treatment, at one month and the end of treatment. The femora were dissected prior to termination. Parameters: Bone 11*β*-HSD type 1 expression (Rattus norvegicus ELISA Kit, USCN Life Science Inc) Serum corticosterone and 11-dehydrocorticosterone levels (ELISA Kit, MyBioSource Inc)	No significant changes of serum corticosterone level between PS treated group and Adx control group. PS group showed significant decreased of femur corticosterone level compare to Adrx group. No significant changes of 11 *β*-HSD type 1 protein expression in the femoral bone between all groups.	Aqueous extract of *Piper sarmentosum* is a potential 11*β*-HSD type 1 inhibitor by switching off the 11 *β*-HSD type 1 reductase activity and protect the bone from glucocorticoid-induced osteoporosis.
**Study 2** **Estai et al. 2012** (24)	Aqueous extract	Bone biomechanical analysis	200 g–250 g of female Sprague-Dawley (*n* = 40)	Rats were randomly grouped into: Sham with normal saline (*n* = 10) OVX with normal saline (*n* = 10) OVX+ERT with conjugated equine estrogen 100 μg/kg (*n* = 10) OVX+PS with PS extracts 125 mg/kg (*n* = 10) Rats were induced for osteoporosis for six week after post-ovariectomised and the treatment start after the induction for next six weeks. All ovariectomised rats undergo closed fracture of femur at the mid-diaphysis. The femora were dissected prior to termination for biomechanically test using Instron Microtester 5848 Model. Parameters: Maximum load, Maximum stress, Young’s modulus and Maximum strain	Maximum load and stress of OVX+PS group was significantly higher compared to the OVX group and no significant difference with Sham and OVX+ERT groups. Young’s modulus OVX+PS group was significantly increased compared OVX group and no significant difference with Sham and OVX+ERT groups. No significant difference of maximum strain among the four groups.	*Piper sarmentosum* supplementation improved the strength and stiffness of bone by restoring the bone biomechanical properties.
**Study 3** **Ramli et al. 2012** (36)	Aqueous extract	Bone biochemical analysis	220 g–250 g of three months male Sprague-Dawley (*n* = 40)	Rats were randomly grouped to: Baseline (*n* = 8) Sham with olive oil 0.05 mL/100 g (*n* = 8) Adrx with dexamethasone, 120 μg/kg (*n* = 8) Adrx+GCA with dexamethasone, 120 μg/kg and GCA 120 mg/kg (*n* = 8) Adrx+PS with dexamethasone, 120 μg/kg and PS extract 125 mg/kg (*n* = 8) Rats were treated for two months after two weeks of adrenalectomy. Femora were dissected at the end of treatment for biochemical tests. Parameters: 11*β*-HSD type 1 dehydrogenase activity 11*β*-HSD type 1 expression	11*β*-HSD type 1 dehydrogenase activity of Adrx+GCA and Adrx+PS groups were not significant difference and both of them were significantly higher than Adx control group. Adrx+PS group was significantly reduced 11*β*-HSD type 1 expression lower than Adrx control group and at par with Sham group.	The increase in dehydrogenase activity of *Piper sarmentosum* extract lead to reduction of active glucocorticoid in bone, thereby protecting bone from glucocorticoid-induced osteoporosis.
**Study 4** **Estai et al. 2011** (25)	Aqueous extract	Bone microarchitecture analysis	200 g–250 g of female Sprague-Dawley (*n* = 24)	Rats were randomly grouped into: Sham with normal saline (*n* = 6) OVX with normal saline (*n* = 6) OVX+ERT with conjugated equine estrogen 100 *μ*g/kg (*n* = 6) OVX+PS with PS extracts 125 mg/kg (*n* = 10) Rats were induced for osteoporosis for six weeks after post-ovariectomised and the treatment start after the induction for next six weeks. All ovariectomised rats undergo closed fracture of femur at the mid-diaphysis. The femora were dissected prior to termination for bone callous evaluation by X-ray and computerised tomographic (CT) scan. Parameters: Bone callous volume, Fracture healing stages	Sham, OVX+ERT, and OVX+PS were not significantly different in callous volume and they are significantly lower than OVX control group. OVX+PS groups showed significant increase fracture healing than OVX group and significantly similar with Sham group.	*Piper sarmentosum* enhance callus maturity by decreasing the callus axial volume increasing the fracture healing score that indicate soft callous volume had been replaced by woven bone during the healing process.
**Study 5** **Estai et al. 2011** (31)	Aqueous extract	Bone histological analysis	200 g–250 g of female Sprague-Dawley (*n* = 24)	Rats were randomly grouped into: Sham with normal saline (*n* = 6) OVX with normal saline (*n* = 6) OVX+ERT with conjugated equine estrogen 100 μg/kg (*n* = 6) OVX+PS with PS extracts 125 mg/kg (*n* = 6) Rats were induced for osteoporosis for six weeks after post-ovariectomised and the treatment start after the induction for next six weeks. All ovariectomised rats undergo closed fracture of femur at the mid-diaphysis. The femora were dissected prior to termination for histological analysis by using H&E staining and Allen’s grading system. Parameter: Fracture healing scores	Median fracture healing score of OVX+PS group was higher than OVXC. No significant difference between OVX+PS with and Sham OVX+ERT groups. Fracture callus stained with H&E of Sham and OVX+PS were identical.	*Piper sarmentosum* extracts able to induce endochondral ossification and accelerating the replacement of soft callus by hard callus (mature callus) as well as preventing osteoporotic changes.
**Study 6** **Ima-Nirwana et al. 2009** (40)	N/A	Bone biochemical analysis	200 g–250 g of three months male Sprague-Dawley (*n* = 40)	Rats were randomly grouped into: Baseline (*n* = 8) Sham with olive oil and distilled water (*n* = 8) Adrx+GCA with dexamethasone, 120 μg/kg and GCA 240 μg/kg (*n* = 8) Adrx+PS with dexamethasone, 120 μg/kg and PS extract 125 mg/kg (*n* = 8) Adrx with dexamethasone, 120 μg/kg (*n* = 8) The treatments were given for eight weeks and serums were collected prior to the termination. Parameters: Serum cortisol level, Plasma pyridinoline level	Serum cortisol level of Adx+PS and GCA groups were maintained at Sham group level. Plasma pyridinoline of Adrx+PS and Adrx+GCA groups were lower than Adx control group, No significant different in serum osteocalcin level for all groups.	*Piper sarmentosum* was effective as GCA in preventing the increased in cortisol level in adrenalectomised rats.

**Table 2:** Review of the effects *Eurycoma longifolia* (EL) on the bone study

	Type of extraction	Type of study	Sample/populations	Methodology	Results	Comments/outcomes
**Study 7** **Azri et al. 2016** (35)	N/A	Bone microarchitecture analysis	300 g–450 g of male Wistar rats (*n* = 48)	Rats were randomly grouped into: Sham with olive oil and normal saline (*n* = 12) ORX with olive oil and normal saline (*n* = 12) ORX+TEN with testosterone 7 mg/kg once a week (*n* = 12) ORX+EL with EL extracts 15 mg/kg daily (*n* = 12) Osteotomy was done after two weeks of osteoporosis developed on metaphysis area using pulsed ultrasound and were fixed with plate and screws. The rats were treated for six weeks and tibiae were dissected for micro-CT (*μ*CT 80 scanner, Scanco Medical) assessment. Parameters: 3D image of bridging cortices Fraction of mineralised tissue of bridging cortices Bone mineral density of bridging cortices Soft callus volume	3D images of ORX+EL show better bridging cortices connections compared than ORX but more inferior to Sham and ORX+TEN groups. ORX+EL have higher fraction of mineralised callus than ORX but not significantly difference. No significant different of soft callus volume of ORX+EL with the other groups.	*Eurycoma longifolia* has shown a better improvement in mineralised tissue of bridging cortices which an important parameter to assess fracture healing level. It showed some potential in promoting fracture healing.
**Study 8** **Azri et al. 2015** (18)	N/A	Bone biomechanical analysis	300 g–450 g of male Wistar rats (*n* = 48)	Rats were randomly grouped into: Sham with olive oil and normal saline (*n* = 12) ORX with olive oil and normal saline (*n* = 12) ORX+TEN with testosterone enanthate 7 mg/kg once a week (*n* = 12) ORX+EL with EL extract 15 mg/kg daily (*n* = 12) Osteotomy was done after two weeks of osteoporosis developed on metaphysis area using pulsed ultrasound and were fixed with plate and screws. The rats were treated for six weeks and tibiae were dissected for biomechanical analysis by using Instron Microtester 5848 Model. Parameters: Maximum load, Maximum stress, Maximum strain, Young’s modulus	ORX+EL groups not significantly improve osteoporotic bone strength compared with negative control.	*Eurycoma longifolia* did not significantly improve the bone callous strength. This is might due to the low dosage of *Eurycoma longifolia* extracts was used or mechanism of testosterone production is failed in the absence of testes.
**Study 9** **Ramli et al. 2012** (41)	Aqueous extract	Bone microarchitecture analysis	300 g–400 g of 10–12 months male Sprague-Dawley (*n* = 48)	Rats were randomly grouped into: Sham (*n* = 8) ORX (*n* = 8) ORX+TEN with testosterone enanthate 7 mg/kg (*n* = 8) ORX+EL30 with EL extracts 30 mg/kg (*n* = 8) ORX+EL60 with EL extracts 60 mg/kg (*n* = 8) ORX+EL90 with EL extracts 90 mg/kg (*n* = 8) Rats were treated for 6 weeks and femora were dissected for micro-CT assessment (*μ*CT 80 scanner, Scanco Medical). Parameters: Bone volume, Connectivity density, Trabecular thickness, Trabecular separation	Trabecular network and bone volume of EL treatment groups were no significant difference with the negative control group.	Supplement of *Eurycoma longifolia* failed to preserve the bone volume and connectivity.
**Study 10** **Saadiah et al. 2012** (45)	Aqueous extract	Bone biomechanical and biochemical analysis	10–12 months of male Sprague-Dawley (*n* = 40)	Rats were randomly grouped into: Sham with olive oil (*n* = 8) ORX with olive oil (*n* = 8) ORX+TEN with testosterone diluted in olive oil 8 mg/kg (*n* = 8) ORX+TEN+EL with testosterone diluted in olive oil 4 mg/rat and EL extracts 15 mg/kg (*n* = 8) ORX+EL with El extracts 15 mg/kg and olive oil (*n* = 8) The rats were treated for 6 weeks. The blood samples were collected before and after the treatment. The tibiae were dissected for biomechanical analysis using Instron Microtester 5848 Model. Parameters: Bone strength Maximum load, Maximum stress, Maximum strain, Young’s modulus Bone markers analysis Serum Osteocalcin level (Rat Osteocalcin ELISA Kit) CTX-1 level (RatlapsTM ELISA CTX-1 Kit)	Post-treatment osteocalcin level of ORX+EL, ORX+TEN+EL were significantly lower than their pretreatment and post-treatment, and also lower compared to osteocalcin level of Sham group. No significant different between post-treatment osteocalcin of ORX+TEN, ORX+EL and ORX+TEN+EL groups. CTX level of post-treatment of OVX+TEN and ORX+TEN+EL were significantly lower than the pre-treatment level. CTX post-tretment level of ORX+EL were lower compared to pre-treatment level but not significantly different. No significantly different of biomechanical groups for all groups except ORX+TEN+EL was significantly higher than compared to ORXC group.	The combination of testosterone and *Eurycoma longifolia* was able to significantly lower both formation and resorption maker which mean it is more effective instead of using testosterone and *Eurycoma longifolia* alone.
**Study 11** **Shuid et al. 2012** (37)	Aqueous extract	Bone biomechanical biochemical and molecular analysis	250 g–300 g of 10 months male Sprague-Dawley (*n* = 32)	Rats were randomly grouped into: Sham (*n* = 8) ORX (*n* = 8) ORX+TEN with testosterone diluted in olive oil 8 mg/kg (*n* = 8) ORX+EL with EL extracts 15 mg/kg (*n* = 8) Blood sample were collected before and after six weeks of treatment and both tibia and femur bones were dissected for bone biomechanical and gene expression determination. Parameters: Bone marker analysis Serum osteocalcin level (Rat Osteocalcin ELISA Kit) Serum CTX-1 level (RatlapsTM ELISA CTX-1 Kit) Bone strength Maximum load, Maximum stress, Maximum strain, Young’s modulus Bone gene expression RANKL, OPG and MCSF gene expression	There was significant reduction in the post-treatment level of CTX-1 level of ORX+EL and ORX+TEN groups compared to their respective pre-treatment levels. There were no significant difference of bone strength between all the groups. RANKL and M-CSF gene expression of tibial bones for all the groups were not significantly different from each other. But EL supplementation was able to increase the OPG expression back to Sham level.	Since *Eurycoma longifolia* was able to elevated OPG expression back to Sham level, it is capable to suppress bone resorption and osteoclastogenetic process induced by androgen deficiency osteoporosis.
**Study 12** **Ariff et al. 2012** (32)	Aqueous extract	Bone histomorphometry analysis	370 g–500 g of 12 months male Sprague-Dawley (*n* = 36)	Rats were randomly grouped into: Normal control, NC (*n* = 8) NC+EL with EL extracts 15 mg/kg Sham (*n* = 8) ORXC (*n* = 8) ORX+TEN with testosterone diluted in olive oil 8 mg/kg (*n* = 8) ORX+EL with EL extracts 15 mg/kg (*n* = 8) Blood sample were collected before and after six weeks of treatment for testosterone level determination and femur were taken out for bone histomorphometry analysis by using Osteo-Bed Bone Embbeding Kit and Von Kassa staining method. Parameters: Trabecular volume, Trabecular thickness, Trabecular number, Trabecular separation	No significant different of histomorphometry result between ORX+EL and ORX+EL groups.	*Eurycoma longifolia* failed to elevate testosterone production even though in normal rat due the possibility that *Eurycoma longifolia* only act during absence of testosterone. It concludes EL unable to preserve bone connectivity.

**Table 3:** Review of the effects *Labisia pumila* (LP) on the bone study

	Type of extraction	Type of study	Sample/populations	Methodology	Results	Comments/outcomes
**Study 13** **Nadia et al. 2017** (38)	Aqueous, methanol and ethanol extracts	Bone microarchitecture analysis	200 g–250 g of three to five months female Sprague-Dawley (*n* = 48)	Rats were randomly grouped into: Sham (*n* = 8) OVX (*n* = 8) OVX+ERT with Premarin estrogen 64.5 μg/kg (*n* = 8) OVX+LP_aq_ with LP aqueous extracts 100 mg/kg (*n* = 8) with LP methanol extracts OVX+LP_met_ 100 mg/kg (*n* = 8) OVX+LP_et_ with LP ethanol extracts 100 mg/kg (*n* = 8) Rats were treated for nine weeks. Femora were dissected out for micro-CT (*μ* CT 80 scanner, Scanco Medical) analysis. Parameters: Bone volume, Bone connectivity density, Trabecular thickness, Trabecular separation, Structure model index (SMI), Degree of anisotropy (DA)	OVX+LP_aq_ group was significantly higher bone volume compare to OVX group and restored up to sham level. No significant different of trabecular thickness between all the groups. Trabecular separation of all treated groups including Sham were significantly lower than OVX group. Trabecular number of OVX+LP_aq_ significantly higher than OVX group and at par with OVX+ERT and Sham groups. Connectivity density OVX+LPaq and OVX+LPmet groups were significantly higher than OVX group and no significant different with OVX+ERT and Sham groups No significant different of DA value between all groups. OVX+LP_aq_ was significant lower SMI than OVX group.	Aqueous extracts of *Labisia pumila* was shown to be the most effective extracts against osteoporosis which mostly result nearly or better than Sham.
**Study 14** **Nadia et al. 2015** (16)	N/A	Bone biomechanical analysis	200 g–250 g of four to five months female Sprague-Dawley (*n* = 96)	Rats were randomly grouped into: Baseline (*n* = 6) Sham (*n* = 18) OVX (*n* = 18) OVX+ERT with Premarin estrogen 64.5 μg/kg (*n* = 18) OVX+LP20 with LP extracts 20 mg/kg (*n* = 18) OVX+LP100 with LP extracts 100 mg/kg (*n* = 18) Rats treated after two week of osteoporosis induction, were subdivided into three weeks, six weeks and nine weeks groups of treatment. The femora were dissected and tested for bone strength using Instron Microtester 5848 Model. Parameters: Maximum load, Displacement, Maximum stress, Maximum strain, Young’s modulus	Maximum load of six weeks and nine weeks of OVX+LP100 was significantly higher than OVX groups of corresponding groups. OVX+LP20 also showed significantly higher maximum load than OVX group after six weeks of treatment. All treated groups showed a significant higher displacement than OVXC group after six weeks of treatment and after nine weeks of treatment OVX+LP100 group result significantly higher than OVX+ERT. OVX+LP20 and OVX+LP100 groups showed significant high stiffness and stress than OVX group after six weeks and nine weeks of treatment. No significant different of strain between the all groups for each week. After nine weeks of treatment, OVX+LP20 and OVX+LP100 groups significantly increasing in Young’s modulus compared to baseline and OVXC groups.	*Labisia pumila* supplementation at dosage of 100 mg/kg for nine weeks of treatment was found to be more effective than ERT in maintaining bone strength.
**Study 15** **Nadia et al. 2014** (39)	N/A	Bone microarchitecture analysis	200 g–250 g of three to five months female Sprague-Dawley (*n* = 96)	Rats were randomly grouped into: Baseline (*n* = 6) Sham (*n* = 18) OVX (*n* = 18) OVX+ERT with Premarin estrogen 64.5 μg/kg (*n* = 18) OVX+LP20 with LP extracts 20 mg/kg (*n* = 18) OVX+LP100 with LP extracts 100 mg/kg (*n* = 18) Rats treated after two week of osteoporosis induction, were divided into three groups, three weeks, six weeks and nine weeks. Femora were taken out for micro-CT (*μ*CT 80 scanner) analysis at metaphysal area with source energy 70 KVp and 114 *μ*A, 10 *μ*m high resolution, 0.5 mm filter used and 200 slices of ROI. Parameters: 3D image microarchitecture, bone volume, connectivity density, trabecular thickness, trabecular separation, and trabecular number	OVX+LP 100 group was the densest trabecular network followed by OVX+LP20 group which was denser than Sham and baseline groups. All LP treated groups were significantly increased bone volume and significantly decreased trabecular separation after six and nine weeks of treatment compared to OVX group. 100 mg/kg of nine weeks showed the lowest trabecular separation. All LP treated groups were significantly increased in connectivity density and trabecular number after nine weeks of treatment compared to OVX group. No significant different of trabecular thickness between all groups.	*Labisia pumila* supplementation has potential in improving trabecular bone microarchitecture and 100 mg/kg LP administration for nine weeks treatment showed the best result in reversing ovariectomy-induced osteoporosis.
**Study 16** **Nadia, Shuid 2014** (55)	N/A	Bone biochemical analysis	200 g–250 g of three to five months female Sprague-Dawley (*n* = 96)	Rats were randomly grouped into: Baseline (*n* = 6) Sham (*n* = 18) OVXC (*n* = 18) OVX+ERT with Premarin estrogen 64.5 μg/kg (*n* = 18) OVX+LP20 with LP extracts 20 mg/kg (*n* = 18) OVX+LP100 with LP extracts 100 mg/kg (*n* = 18) Rats then were subdivided into groups, three weeks, six weeks and nine weeks groups of treatment. The femora were dissected prior to termination bone oxidative status analysis. Parameters: Superoxide dismutase (SOD) expression (Superoxide Dismutase Assay Kit) Glutathione peroxide (GPx) expression (Glutathione Peroxidese Assay Kit) Malondialdehyde (MDA) expression (TBARS Assay Kit)	OVX+LP20 and OVX+LP100 groups recorded significantly higher of SOD level than OVX group of corresponding week after six weeks and nine weeks treatment. While OVX+LP20 of six weeks treatment group showed the best SOD level that was significantly higher than OVX+ERT group. OVX+LP100 group was significantly higher GPx level than baseline and OVXC groups at nine weeks of treatment. MDA level of OVX+LP100 and OVX+LP20 groups were significant lower than OVX and OVX+ERT groups after nine weeks of treatment.	*Labisia pumila* has potential to increase anti-oxidative enzymes and reduce the oxidative stress in estrogen-deficiency postmenopausal rats. Flavonoids and phenolic compound which are anti-oxidative component found in *Labisia pumila* that resemble feature of estrogen which allowed them to regulate osteoblast activity.
**Study 17** **Fathilah et al. 2013** (56)	Aqueous extract	Bone molecular analysis	200 g–250 g of three months female Wistar rats (*n* = 32)	Rats were randomly grouped into: Sham with deionised water (*n* = 10) OVX with deionised water (*n* = 10) OVX+ERT with conjugated estrogen Premarin 64.5 μg/kg (*n* = 10) OVX+LP with LP 17.5 mg/kg (*n* = 10) Rats were orally treated for eight weeks. Femora were dissected prior to termination for bone gene expression evaluation using branch DNA technique. Parameters: RANKL, OPG, MCSF and BMP-2 gene expression.	The level of RANKL, OPG and BMP-2 gene expression of OVX+LP group were not significantly difference with Sham and OVX+ERT groups. No significant different of MCSF gene expression between all the groups.	*Labisia pumila* was able to reduce ovariectomy-induced elevation of RANKL gene expression and increase the declining OPG and BMP-2 expressions.
**Study 18** **Fathilah et al. 2012** (33)	Aqueous extract	Bone histomorphometry analysis	200 g–250 g of three months female Wistar rats (*n* = 32)	Rats were randomly grouped into: Sham with deionised water (*n* = 10) OVX with deionised water (*n* = 10) OVX+ERT with conjugated estrogen Premarin 64.5 μg/kg (*n* = 10) OVX+LP with LP 17.5 mg/kg (*n* = 10) Rats were orally treated for eight weeks. Femora were dissected for bone histomorphometry by using Osteo-Bed Bone Embedding Kit and Von Kassa staining method. Parameter bone histomorphometry: Structural parameter trabecular volume, thickness, number and separation Static parameters Osteoblast surface, osteoclast surface, eroded surface, osteoid volume and osteoid surface Dynamic parameters Single-labeled surface, double-labeled surface, mineralising surface, mineral apposition rate and bone formation rate	All the structural parameters of OVX+LP group was significantly higher than OVXC group and no statistical differ with OXV+ERT group. While there was no significantly different in static parameter between OVX+LP and Sham and OVX+ERT groups. OVX+LP and OVX+ERT were significantly better of dynamic parameters than Sham and OVX group.	*Labisia pumila* has potential alternative to replace estrogen replacement therapy in order to diminish the side effect of hormonal replacement. *Labisia pumila* contain phtoestrogenic actions that thought to interact with estrogen receptor for bone regulation and also found able to suppress cytokine which promote bone resorption.
**Study 19** **Fathilah et al. 2012** (54)	Aqueous extract	Bone biomechanical analysis	200 g–250 g of three months female Wistar rats (*n* = 32)	Rats were randomly grouped into: Sham with deionised water (*n* = 10) OVX with deionised water (*n* = 10) OVX+ERT with conjugated estrogen Premarin 64.5 μg/kg (*n* = 10) OVX+LP with LP 17.5 mg/kg (*n* = 10) Rats were orally treated for eight weeks. Femora bones were taken out for bone strength analysis using Instron Universal Testing Machine. Parameters: Load, stress, strain and Young’s modulus	All parameters indicated OVX+LP and OVX+ERT groups were significantly similar to each other and significantly higher than sham and OVX groups.	*Labisia pumila* has potential to act better and safer than estrogen replacement therapy in preserving the bone strength and preventing estrogen-deficient osteoporosis.
**Study 20** **Nadia et al. 2012** (53)	N/A	Bone biomechanical analysis	Three to four months female Sprague-Dawley (*n* = 96)	Rats were randomly grouped into: Baseline (*n* = 6) Sham (*n* = 18) OVX (*n* = 18) OVX+ERT with Premarin estrogen 64.5 μg/kg (*n* = 18) OVX+LP20 with LP extracts 20 mg/kg (*n* = 18) OVX+LP100 with LP extracts 100 mg/kg (*n* = 18) Rats treated after two week of osteoporosis induction, were subdivided into three weeks, six weeks and nine weeks groups of treatment. Prior to termination, femora were taken out for micro- CT (*μ*CT 80 scanner, Scanco Medical) analysis. Parameters: Bone volume, connectivity density, trabecular thickness, trabecular separation and trabecular number	All parameters recorded that OVX+LP100 was more effective than ERT after nine weeks of treatment.	*Labisia pumila* was able to restore trabecular network back to normal state and turn it as a potential of effective treatment in reversing ovariectomy-induced osteoporosis.
**Study 21** **Shuid et al. 2011** (51)	Aqueous extract	Bone biochemical analysis	Three months female Wistar rats (*n* = 32)	Rats were randomly grouped into: Sham with deionised water (*n* = 10) OVXC with deionised water (*n* = 10) OVX+ERT with conjugated estrogen Premarin 64.5 μg/kg (*n* = 10) OVX+LP with LP 17.5 mg/kg (*n* = 10) Rats were orally treated for eight weeks. Blood was collected before and after the treatment for bone resorption marker test. The fifth lumbar vertebrate was dissected out for bone calcium test. Parameters: Bone marker analysis Serum osteocalcin level (Rat Osteocalcin ELISA Kit) Serum CTX-1 level (RatlapsTM ELISA CTX-1 Kit) Bone calcium content (Atomic Absorption Spectrophotometer)	Osteocalcin and CTX-1 level of compared to OVX+LP was similar with OVX+ERT and Sham groups. The bone calcium content of OVX+LP no significantly difference with the OVX groups.	Even though *Labisia pumila* supplementation unable to preserve bone calcium content back to normal, it was able to reduce bone resorption marker and promoting bone formation marker. Thus LP is comparable with estrogen replacement therapy.
